# Replication of the Association of a *MET* Variant with Autism in a Chinese Han Population

**DOI:** 10.1371/journal.pone.0027428

**Published:** 2011-11-08

**Authors:** Xue Zhou, Yang Xu, Jia Wang, Hongbo Zhou, Xian Liu, Qasim Ayub, Xuelai Wang, Chris Tyler-Smith, Lijie Wu, Yali Xue

**Affiliations:** 1 Department of Children's and Adolescent Health, Public Health College of Harbin Medical University, Harbin, Heilongjiang, People's Republic of China; 2 The Wellcome Trust Sanger Institute, Wellcome Trust Genome Campus, Hinxton, Cambridgeshire, United Kingdom; 3 Department of Biochemistry and Molecular Biology, Basic Medical Science College of Harbin Medical University, Harbin, Heilongjiang, People's Republic of China; Tsan Yuk Hospital, Hospital Authority, China

## Abstract

**Background:**

Autism is a common, severe and highly heritable neurodevelopmental disorder in children, affecting up to 100 children per 10,000. The *MET* gene has been regarded as a promising candidate gene for this disorder because it is located within a replicated linkage interval, is involved in pathways affecting the development of the cerebral cortex and cerebellum in ways relevant to autism patients, and has shown significant association signals in previous studies.

**Principal Findings:**

Here, we present new ASD patient and control samples from Heilongjiang, China and use them in a case-control and family-based replication study of two *MET* variants. One SNP, rs38845, was successfully replicated in a case-control association study, but failed to replicate in a family-based study, possibly due to small sample size. The other SNP, rs1858830, failed to replicate in both case-control and family-based studies.

**Conclusions:**

This is the first attempt to replicate associations in Chinese autism samples, and our result provides evidence that *MET* variants may be relevant to autism susceptibility in the Chinese Han population.

## Introduction

Autism is a severe neurodevelopmental disorder of childhood, diagnosed on the basis of impaired social interaction and communication, the presence of rigid and stereotyped behaviours, and disease onset prior to 3 years of age [1]. Autism spectrum disorder (ASD) is a term that encompasses a broader set of conditions, including autistic disorder, sometimes called “classic” autism, Asperger syndrome, and pervasive developmental disorder not otherwise specified (PDD-NOS) [1].

The prevalence of ASD is reported to have increased over time [2], [3]. The current prevalence estimates for ASD range from 90 to more than 100 per 10,000 in the United States [4] and most other European countries [5], [6], [7], [8] with a male:female ratio of 3.3∶1 to 4.5∶1. In China, the prevalence has been reported as ranging from 11.0 to 22.7 per 10,000 in different surveys [9], [10], [11], [12], [13], with a male:female ratio of 1.29∶1 to 7.0∶1.

Autism is a highly heritable disorder, with a heritability of 70 to 90% [14], [15], [16], [17], and enormous efforts in searching for genetic susceptibility loci have been going on for decades [17]. The initial focus was on linkage analysis, and although hundreds of candidate loci were reported, few have been replicated [18]. Genome-wide association studies (GWAS) seeking evidence for association with common SNPs have identified some likely candidate regions, but these can only account for a small proportion of the heritability [17]. Rare CNVs have been implicated as possible causal variants [19], and studies of candidate genes have also achieved some success.

The *MET* gene lies within one of the most prominent linkage intervals, which is also one of the few linkage peaks which have been replicated in independent studies [18]. It codes for the MET receptor tyrosine kinase and is activated by the hepatocyte growth factor/scatter factor (HGF/SF). Although it was initially identified as a proto-oncogene and plays a role in human cancer development [20], MET signalling is also involved in many other processes, such as immune system regulation [21], [22], embryogenesis and in the peripheral organ development and repair, for example, gastrointestinal [23], [24]. Most relevantly, MET and HGF are both implicated in neuronal development, especially in the development of the cerebral cortex and cerebellum, where they show a highly specific expression pattern. Phenotypic characteristics caused by impaired MET/HGF signalling such as modified neuronal migration and disrupted neuronal growth in the cortex, as well as a decreased proliferation of granule cells causing a parallel reduction in the size of the cerebellum, have been seen in the autism patients [25], [26], [27], [28], [29], [30]. These lines of evidence have made *MET* a good autism candidate gene meriting further investigation.

Campbell and colleagues [25] first reported an association of a common promoter variant (G to C, rs1858830) located 20 bp 5′ to the transcription start site of *MET* with autism. Subsequent studies have attempted to replicate the association: the same authors replicated their finding in independent samples in the same report [25] and then in a third European sample [31]. Jackson et al. [32] replicated the association of the rs1858830 C allele in two more samples. Sousa et al. failed to replicate association of rs1858830 in their sample, but did identify another SNP, rs38845 (within 10 kb of rs1858830) as being associated with ASD [33]. Thanseem et al. [34] found association of a third SNP (rs38841; also within 10 kb of rs1858830) in two samples, one Japanese and the other American (AGRE).

There has been little investigation of the genetic basis of autism in the Chinese population, with most studies limited to Western populations. We have collected 361 Chinese core family samples consisting of affected child, mother and father, as well as 44 sporadic patient samples, together with matched controls, to initiate a Chinese autism genetic resource for future research. With these samples, we have begun to test whether the susceptibility genes and SNPs identified in the European population can be replicated in the Chinese population, and here report our initial findings on the association of *MET* SNPs with autism in a Chinese Han sample.

## Results

All 361 trios (both parents and one affected child) and 44 sporadic ASD children, as well as 594 controls (Table 1), were successfully genotyped for rs38845 using an ABI PRISM SNaPshot Multiplex Kit (Materials and Methods, Table 2). The genotype frequencies of rs38845 in both ASD cases and controls were in Hardy-Weinberg equilibrium (χ^2^  = 0.247, p = 0.619 and χ^2^ = 0.702, p = 0.402, respectively). However, four trio genotype sets did not conform to a Mendelian transmission pattern and were excluded from further analysis.

**Table 1:** Characteristics of the control and ASD groups.

	Number of samples	Age (Mean±SD)	sex ratio(male:female)
Trios with ASD children	361	4.67±2.19	310∶51
Autism (trio child)	331	4.72±2.22	282∶49
PDD-NOS (trio child)	30	4.14±1.82	28∶2
Sporadic ASD children	44	5.31±2.53	39∶5
Autism	41	5.03±2.16	37∶4
PDD-NOS	3	9.06±3.87	2∶1
Controls	594	17.37±5.62	489∶105

**Table 2:** Primers and PCR amplification conditions for *MET* SNPs.

Gene and SNP	Primers for specific fragment	Size (bp)	Cycles for PCR amplification	Primers for extension PCR or sequencing	Size (bp)
MET rs1858830	Forward: 3′-GATTTCCCTCTGGGTGGTGC	652	Touchdown PCR: 15 cycles at 94°C for 30 sec, annealing temperature of 70°C for 30 sec, decreased by 0.5°C every cycle and 68°C for 45 sec. 20 cycles at 94°C for 30 sec, 60°C for 30 sec and 68°C for 45 sec. PCR mixtures contained 5% DMSO	Extension primer: 3′-CCTCCGGGTCACCTGCGGCC	20
	Reverse: 3′-CAAGCCCCATTCTAGTTTCG			Sequencing primers1: 5′-GATGCCCGGCTGAGTCACTGGC	
				Sequencing primers2: 5′- TGGTCGCCTGGCGGTGCCTCCG	
MET rs38845	Forward: 3′-CTGATTCTGCCCTCACTTCAG	374	95°C for 10 min, then 28 cycles at 95°C for 60 sec, 56°C for 30 sec and 72°C for 60 sec	Extension primer: 5′-ACTGACTGACAGCCAATGGCTACACTGTCC	30
	Reverse: 3′-AAGTATGTGTTAGTGAGACCGAAA				

The allele frequencies of rs38845 in the controls were 39.7% A and 60.3% G, which are almost as same as in the HapMap healthy Han Chinese samples (40% and 60%, respectively), providing additional confidence in the genotyping results. There was a significant difference in the rs38845 allele frequency between ASD cases (401 cases) and controls (OR = 1.24 (1.03–1.49); p = 0.019). If we only considered the autism cases (371 cases), a slightly more significant difference was found (OR = 1.28 (1.06–1.54), p = 0.010) (Table 3). Our case and control male:female ratios were somewhat different, and even though we do not expect the allele frequency to differ between males and females for a marker located on an autosome, we subsampled our controls (568 out of total 594) to match the case male:female ratio. The association was similar and still significant (OR = 1.26 (1.05–1.52), p = 0.015). We also found a higher frequency of the rs38845 A allele in the cases compared with the controls, as in the previous report [33], so the association is in the same direction. This result shows that the association of the rs38845 A allele with both autism only and ASD can be replicated in Chinese Han samples.

**Table 3:** Statistical analysis of rs38845 and rs1858830 in the Chinese Han case and control samples.

	No. of subjects	Genotype frequencies			Allele frequencies		p value	OR (95% CI)
rs38845		GG	GA	AA	G	A		
Cases (All ASD)	401	126 (0.314)	189 (0.471)	86 (0.215)	441 (0.550)	361 (0.450)	0.019*	1.242 (1.036–1.489)
Cases (autism only)	369	115 (0.312)	171 (0.463)	83 (0.225)	401 (0.543)	337 (0.457)	0.010**	1.275 (1.059–1.535)
Controls	594	218 (0.367)	280 (0.471)	96 (0.162)	716 (0.603)	472 (0.397)		

rs1858830		GG	GC	CC	G	C		
Cases (All ASD)	393	174 (0.443)	168 (0.427)	51 (0.130)	516 (0.656)	270 (0.344)	0.861	1.017 (0.840–1.231)
Controls	570	240 (0.421)	264 (0.463)	66 (0.116)	744 (0.653)	396 (0.347)		

Since we had genotype data from 357 trios, we also carried out a family-based TDT test. Although we saw slight over-transmission of the A allele, it was not significant, probably because of the small sample size (Table 4). To investigate this further, we estimated the minimum number of trios needed for 80% power to detect over-transmission, given the Risk Ratio or RR (1.16, 95% CI: 1.04–1.30) calculated from our case-control analysis. We need 746 or 2,737 trios if we use an RR of 1.2 or 1.1, respectively. The samples size we have will only provide 80% power to detect the association with an RR of at least 1.34. Thus the failure to detect association using the TDT test likely reflects the small sample size.

**Table 4:** Statistical analysis (TDT) of rs38845 and rs1858830 in the Chinese Han trio samples.

rs38845	No. of subjects	G frequency	A frequency	Transmitted	Nontransmitted	statistic test
Trios	357	0.564	0.436	181	171	X^2^ = 0.284, P = 0.59

rs1858830		G	C			
Trios	343	0.644	0.356	153	134	X^2^ = 1.258, P = 0.262

The SNP rs1858830, which lies in a GC-rich region, failed in the SNaPshot genotyping assay. However, 343 out of 361 trios, 393 out of 401 cases and 570 out of 594 controls (in all, 96%) were successfully genotyped using BigDye Sanger sequencing. The genotype frequencies of rs1858830 in both ASD cases and controls were in Hardy-Weinberg equilibrium (χ^2^ = 1.017, p = 0.301 and χ^2^ = 0.264, p = 0.608, respectively), but we failed to find any association in both case-control (Table 3) and TDT analyses (Table 4). We also carried out the same power analysis for this SNP, using the RR or OR calculated from the reported allele frequency differences [25], and the allele frequency observed in our samples. We need 239 cases and 1,037 trios to get 80% power to detect such a strong association with such a high frequency SNP. So our failure to replicate this marker is not because of low power, at least for the case-control association.

## Discussion

Autism is a highly heritable complex disorder and efforts to identify its genetic basis have been going on for decades. Linkage studies showed a strong signal in the 7q21–36 region associated with autism in multiple analyses [18]. The *MET* gene located in the region was considered a good candidate both because of the biological importance of the MET/HGF signalling pathway in autism aetiology [26], [27], [28], [29], [30] and also because of the successful association of *MET* variants with autism in previous studies in European and Japanese populations [25], [31], [32], [33], [34].

There have been very limited previous attempts to study the genetics of autism in China, and few samples have been available (around 200 cases and 300 controls [35]. We therefore started our studies of the genetics of autism in China with a replication study focusing on *MET* variants. We replicated the association of one SNP, rs38845, but not that of another, rs1858830, with autism in our sample collections. Although significant associations for rs38845 were found in both ASD cases and autism only cases, the real association may be with autism only, because we observe a smaller value with this sample. The small size (33 samples) of non-typical autism cases included in the analysis provides insufficient power to test for a signal in these cases separately. We considered the possibility that failed replication of rs1858830 in our sample collection could be due to a different LD structure in the Chinese population. We calculated both D' and r^2^ for these two markers and found that they are not in LD (D' = 0.03 and r^2^ = 0.00) which is actually similar to the European population where D' is 0.21 [33]. This could suggest the presence of genetic heterogeneity, with different variants of the *MET* gene contributing to autism aetiology in different populations.

In an association study, we always need to consider whether or not an association we find could be explained by sample stratification rather than disease status. In our study, four lines of evidence help to rule out stratification. First, we matched the control samples very strictly to the cases, and the sample collection was restricted to one region of China (Heilongjiang Province) and one ethnic group (Han). Although genetic differences between Han from the different parts of China have been reported, they are very homogeneous within each region of the China [36]. Second, the rs38845 allele frequency we found in our control samples was the same as that in the HapMap samples, which are also from the northern part of China, providing direct support for a lack of substantial frequency differences within northern China at this allele. Third, we also carried out a family-based study (TDT analysis), which is not influenced by population stratification. Although we could not confirm the association with this test, this failure was probably due to the small sample size and the availability of only simplex families in China because of the one-child policy; the previous study found a stronger effect of *MET* SNPs in families with >1 affected child; nevertheless, we did see over-transmission of the same allele in the autism-affected children. Finally, rs1858830 did not show any association. With all these lines of evidence, we conclude that the association for rs38845 we have replicated is very likely real.

Here we document what is to our knowledge the first sample collection devoted to autism genetics in China, which aims to reach more than 1000 cases and controls within the next two years. This opens the door to international collaborations for comprehensively understanding the genetic basis of autism in an international context.

## Materials and Methods

### Subjects

Study subjects were recruited from the Children Development and Behavior Research Center (CDBRC), Harbin Medical University, Heilongjiang Province, China, from Jan. 2007 to Dec. 2009. All cases were diagnosed independently by more than two experienced psychiatrists according to the international Diagnostic and Statistical Manual of Mental Disorders, fourth edition (DSM-IV) [17] criteria for autism. All the patients were also assessed using the Childhood Autism Rating Scale (CARS) [37] and autism behavior checklist (ABC) at the same time [38]. Cases with Rett syndrome, tuberous sclerosis, fragile-X syndrome, and any other neurological conditions suspected to be associated with autism were excluded by clinical examination and a molecular genetic test of the *FMR1* gene [39].

361 trios (both parents and one child) with ASD children and 44 sporadic ASD children were collected in the study. General information on the samples included in this study is summarized in Table 1. The 405 ASD children were classified as follows: Autistic Disorder (372 cases), PDD-NOS (33 cases). All the 405 ASD children were of Chinese Han ethnicity and had a mean age of 4.74±2.24 years at the time of diagnosis. 56 were females and 349 were males giving a male:female sex ratio of 6.23∶1. A total of 594 Chinese Han controls were also recruited, comprising 489 males and 105 females with ages from 3 to 24 years for case-control association analysis. We matched the cases and controls based on their ancestral geographical origin (three generations from the same province), life standard (below average, average, above average) and the parents' education level. All control children were examined clinically in the same way and none demonstrated any features of developmental delay or autistic traits assessed using the same criteria.

All parents provided written informed consent for themselves and their children. Ethical permission for this study was obtained from Ethics Committee of Harbin Medical University [approval number HAYIWEILUNSHENZi 2007016]. About 5 ml blood was collected from each patient or control and genomic DNA extracted from 200 ul using a QIAamp DNA Mini Kit (QIAGEN, Germany) according to the manufacturer's instructions.

### SNP genotyping

Two SNPs were genotyped: rs1858830 upstream of the *MET* gene and rs38845 in the first intron, using an ABI PRISM SNaPshot Multiplex Kit as described by Xue et al. [40], except that the initial PCRs to amplify the fragment containing each SNP were done separately. The primers and PCR conditions are summarized in Table 2. The PCR products were then mixed in roughly equal molar amounts, as estimated from their gel band intensities. The extension PCR products were run on an ABI PRISM 3100 Genetic Analyzer and data were viewed and analyzed using GeneScan Analysis Software version 3.5.

The SNP rs1858830 failed in the genotyping assay. The original PCR products were cleaned up twice using a MultiScreen PCRµ96 Filter Plate (Millipore), then sequenced by BigDye Sanger sequencing using two pairs of forward primers two or more times each (downstream of this SNP is a repeat sequence that prevents sequencing using a reverse primer). The sequencing primers are listed in Table 2. Variable positions were flagged by the Mutation Surveyor v. 2.0 software (SoftGenetics) and then confirmed manually.

### Statistical Analysis

Genotypes were coded as GG, GA or AA for rs38845 and GG, GC, CC for rs1858830, and genotype and allele frequencies were calculated by direct counting. Data quality control was performed by checking genotypes for Mendelian consistency within families and testing Hardy-Weinberg equilibrium in both cases and controls using a chi-square test. The case-control association analysis (a chî2 analysis of contingency table) was performed via the web-based tool, SISA (http://www.quantitativeskills.com/sisa/). Both RR and OR were calculated using a multiplicative risk model. A family-based transmission disequilibrium test (TDT) [41], [42] was performed by comparing the transmitted allele and the non-transmitted allele from heterozygous parents to the affected child using Haploview (version 4.2) [43] and a chi-square test. We used the QUANTO program (http://hydra.usc.edu/gxe/) for power calculations.
